# Polysubstance use in a community sample of Black cisgender sexual minority men and transgender women in Chicago during initial COVID-19 pandemic peak

**DOI:** 10.1186/s13011-022-00433-x

**Published:** 2022-01-28

**Authors:** Raymond L. Moody, Yen-Tyng Chen, John A. Schneider, Justin Knox, Liadh Timmins, Hillary Hanson, Kangkana Koli, Mainza Durrell, Jessica Dehlin, Rebecca Eavou, Silvia S. Martins, Dustin T. Duncan

**Affiliations:** 1 Department of Epidemiology, Columbia University Mailman School of Public Health, 772 West 168th Street, New York, NY 10032 USA; 2 Department of Psychiatry, Columbia University Irving Medical Center, 1051 Riverside Drive, New York, NY 10032 USA; 3 HIV Center for Clinical and Behavioral Studies, New York State Psychiatric Institute and Columbia University, 1051 Riverside Drive, New York, NY 10032 USA; 4 Department of Public Health, William Paterson University of New Jersey, 300 Pompton Road, Wayne, New Jersey, 07470 USA; 5 Chicago Center for HIV Elimination, University of Chicago, 5837 South Maryland Avenue, Chicago, IL 60637 USA; 6 Department of Public Health Sciences, University of Chicago, 5841 South Maryland Avenue, MC2000, Chicago, IL 60637 USA; 7 Department of Medicine, University of Chicago, 5841 South Maryland Avenue, MC6092, Chicago, IL 60637 USA; 8 Department of Sociomedical Sciences, Columbia University Mailman School of Public Health, 772 West 168th Street, New York, NY 10032 USA; 9 Survey Lab, University of Chicago, 5841 South Maryland Avenue, Chicago, IL 60637 USA

**Keywords:** COVID-19, Polysubstance use, Black, Sexual minority men, Transgender women

## Abstract

**Background:**

In response to COVID-19, the city of Chicago issued stay-at-home orders, which began on March 20, 2020, and restrictions meant to “flatten the curve” remained in effect until June 2, 2020. On June 3, 2020, Chicago entered the reopening phase. This study compares rates of polysubstance use by COVID-19 lockdown phase and across sociodemographic characteristics in a Chicago-based sample of Black cisgender sexual minority men (SMM) and transgender women.

**Method:**

Data come from the Neighborhood and Networks (N2) cohort, an ongoing study of Black cisgender SMM and transgender women living in Chicago. Participants (*N* = 226) completed a survey between April 20, 2020, and July 30, 2020, during the initial peak of the COVID-19 pandemic in Chicago. We conducted chi-square tests of independence and modified Poisson regression models with robust error variance and estimated adjusted prevalence ratios.

**Results:**

Alcohol and marijuana were the most used substances, with 73.5% reporting at least one drinking day and 71.2% of the sample reporting marijuana use in the past 14 days. Tobacco was used by 41.6% of the sample, and illegal drug use, which does not include marijuana, was reported by 17.7% of the sample. Substance use was consistently associated with the use of other substances. As such, polysubstance use (i.e., using two or more substances) was common in this sample (63.7%). Few sociodemographic differences emerged, and substance use was not associated with lockdown phase.

**Conclusion:**

Substance use, including polysubstance use, was high in our sample of Black SMM and transgender women during the initial peak of the COVID-19 pandemic. Continued monitoring is needed given the duration of the COVID-19 pandemic and the negative health consequences associated with substance use in this population.

**Supplementary Information:**

The online version contains supplementary material available at 10.1186/s13011-022-00433-x.

## Background

COVID-19 has led to widespread closures of businesses, schools, public transportation, and gatherings to reduce virus transmission. The city of Chicago (the site of the current study) issued stay-at-home orders that began on March 20, 2020, and most restrictions remained in effect until June 2, 2020. On June 3, 2020, Chicago entered the reopening phase, allowing most businesses to reopen at reduced capacity. Despite the rapid development and emergency use authorization of COVID-19 vaccines, new variants have prolonged the COVID-19 pandemic and have raised concern that restrictive mitigation strategies may again be necessary [1]. There is interest in understanding the impacts of this public health response on other health outcomes, such as substance use.

Preliminary evidence suggests that COVID-19 restrictions may increase substance use [2–4]. For example, an international study conducted in April 2020, after most US states issued stay-at-home orders, found that 35.0% of adults were using substances to cope, where 35.6% of adults who drank alcohol reported drinking more than usual, and 55.1% of adults who used marijuana reported smoking more than usual [5]. Several studies of adults in the US have demonstrated sharp increases in the sales and usage of alcohol and marijuana during the early weeks of the COVID-19 pandemic [3, 6–8]. In one study of US adults, average drinks per day increased by 29.0%, drinking that exceeded drinking limits increased by 20.0%, and binge drinking increased by 21.0% following the initiation of stay-at-home orders [7]. This study noted that the increase in drinking that exceeded drinking limits was significantly higher among Black, non-Hispanic respondents compared to White, non-Hispanic respondents. A study of sexual minority men (SMM) in the US found that 26.0% reported increased alcohol use, and 9.9% reported increased drug use since the COVID-19 pandemic began [9]. However, some studies involving SMM and transgender women suggest that substance use prevalence decreased but substance use frequency increased among those who continued using early in the COVID-19 pandemic [10, 11].

A defining characteristic of COVID-19 is its disproportionate impact on marginalized groups [12]. This is consistent with research indicating sexual and gender minorities experience a disproportionate burden of adverse health outcomes, including substance use [13, 14]. Thus far, we know little about substance use rates among Black SMM and transgender women during the COVID-19 pandemic despite evidence highlighting disproportionate impacts of COVID-19 on Black communities [12, 15]. Preliminary data suggests that the COVID-19 pandemic has likely worsened income, employment, and housing disparities affecting racial and ethnic minorities [16, 17]. Moreover, individuals living at the intersection of multiple marginalized identities may experience different health risks compared to those with less marginalized status [18].

Polysubstance use, defined as using two or more substances, remains a health risk factor for sexual and gender minorities, with less known among Black SMM and transgender women [13, 14, 19, 20]. Polysubstance use has been identified as part of a syndemic affecting sexual and gender minorities, contributing to the burden of disease, including HIV among this population [19, 21]. As such, understanding the impact of the COVID-19 pandemic on polysubstance use among Black SMM and transgender women remains an important area of research. The objective of the current study was to characterize substance use, including polysubstance use, during the initial peak of the COVID-19 pandemic in a Chicago-based cohort of Black cisgender SMM and transgender women. We compare rates of substance use during the restrictive phase (March 20, 2020, to June 2, 2020) to rates during the reopening phase (June 3, 2020, to July 31, 2020) and across sociodemographic characteristics.

## Methods

### Participants and procedures

Data come from the ongoing Neighborhood and Networks (N2) cohort study of Chicago-based Black cisgender SMM and transgender women [22]. Enrollment in the cohort began in February 2018 and 412 participants completed the baseline assessment. To be eligible for N2, participants had to: (a) identify as Black or African American; (b) be assigned male sex at birth; (c) reside in Chicago; (d) not have plans to move outside Chicago during the study; (e) report at least one sexual encounter with a man or transgender woman in the past year; (f) be willing to wear a GPS device; and (g) be between 16 and 34 years old.

Beginning April 20, 2020, we asked participants to complete a supplementary COVID-19 survey via Zoom platform or by phone [23]. Data collection for the survey ended on July 30, 2020, and we included the 226 baseline N2 participants who completed the survey in these analyses. The interview lasted 40 min on average, and participants were compensated $35. The study protocol was reviewed and approved by the Biological Sciences Division/University of Chicago Medical Center and the Columbia University Mailman School of Public Health Institutional Review Boards.

### Measures

#### Substance use

Participants provided retrospective reports of substance use over the past 14 days. For alcohol, participants reported how many days they had an alcoholic beverage and how many days they had five or more drinks on the same occasion. Participants also reported if they have been drinking more, fewer, or the same number of alcoholic beverages and if they have been drinking stronger, weaker, or the same strength alcoholic beverages since the stay-at-home order. For tobacco, participants reported how many days they smoked all or part of a cigarette and how many cigarettes, on average, they smoked on smoking days. Lastly, participants indicated if they used marijuana or any illegal drugs (i.e., crack-cocaine; powder-cocaine; methamphetamine; ecstasy/MDMA; GHB/GBL; ketamine; and heroin).

#### Sociodemographic characteristics

Sociodemographic characteristics were assessed in the N2 baseline survey and linked with the COVID-19 survey. Participants reported their age, gender identity, sexual orientation, relationship status, education, employment status, homelessness, and income. As part of the COVID-19 survey, participants reported if they experienced a loss of income, health insurance, or place to stay due to the COVID-19 pandemic.

### Analysis

We calculated a *lockdown phase* variable based on a participant’s survey date. Using data from the Illinois Department of Public Health, we defined the restrictive phase as the phase ranging from March 20, 2020, to June 2, 2020, and the reopening phase ranging from June 3, 2020, to July 31, 2020. We calculated variables for *alcohol us*e, *tobacco use, marijuana use,* and *illegal drug use* indicating if a participant used the substance (0 = no use; 1 = use), and a *polysubstance use* variable based on the number of substances used from the alcohol, tobacco, marijuana, and illegal drug variables (0 = less than two substances; 1 = two or more substances). We conducted chi-square tests of independence to examine differences in substance use by lockdown phase and across sociodemographic characteristics. To examine the effects of lockdown, sociodemographic characteristics, and substance use on our substance use outcomes, we ran modified Poisson regression models with robust error variance and estimated adjusted prevalence ratios (APRs) because of the high prevalence of substance use in the sample [24, 25]. To account for multiple comparisons and reduce the likelihood of Type 1 error in our analyses, we utilized a more conservative threshold (*p* < .01) when interpreting our analyses. We ran analyses in SPSS (version 27).

## Results

A total of 226 participants participated in this survey and we present the sociodemographic characteristics of the sample in Table 1. The mean age of the sample was 25.72 (SD = 4.03). At their pre-COVID baseline assessment, participants predominately identified as male (88.1%), gay (58.0%), single (61.1%), had a high school education or more (89.8%), were unemployed (57.5%), earned less than $20,000 per year (62.5%), and had not experienced homelessness in the past 12-months (74.8%). At the time of their COVID-19 survey, 75.7% reported losing/not having an income, 21.6% reported losing/not having health insurance, and 24.3% reported losing/not having a place to stay.

**Table 1:** Sociodemographic characteristics of the sample (*N* = 226)

	Total	
	n	%
Phase of Lockdown		
Restrictive Phase	77	34.1
Reopening Phase	149	65.9
Age		
16–22	51	22.7
23–24	49	21.8
25–28	63	28.0
29–36	62	27.6
Gender		
Male	196	86.7
Trans Feminine/Other	30	13.3
Sexual Orientation		
Gay	131	58.0
Bisexual/Straight/Something Else	95	42.0
Relationship Status		
In Relationship	138	61.1
Single	88	38.9
Education		
Less than HS Diploma	23	10.2
HS Diploma	203	89.8
Employment		
Employed	130	57.5
Unemployed	96	42.5
Income		
Less than $20 K	140	62.5
$20 K plus	84	37.5
Homelessness (past 12 months)		
No	169	74.8
Yes	57	25.2
Income Status during COVID-19 Pandemic		
No Income Before / Lost Income	171	75.7
Had Income and did not lose it	55	24.3
Health Insurance Status during COVID-19 Pandemic		
No Insurance Before / Lost Insurance	48	21.6
Had Insurance and did not lose it	174	78.4
Housing Status during COVID-19 Pandemic		
No place to stay before / Lost place to stay	55	24.3
Had place to stay and did not lose it	171	75.7

**Fig. 1:**
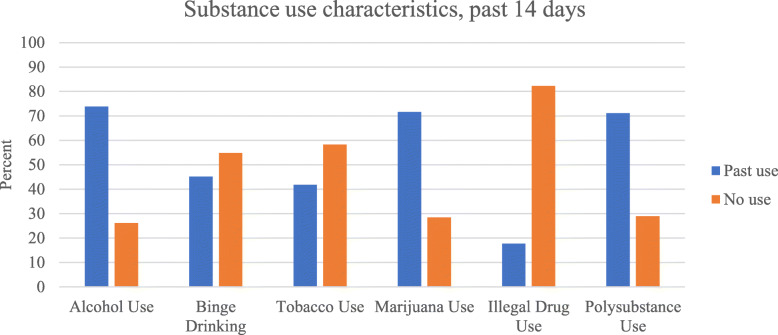
Overall percentage of sample who reported alcohol use, binge drinking, tobacco use, marijuana use, and polysubstance use in the past 14 days. Note: *N* = 226. Binge drinking is only among participants who reported at least one drinking day in the past 14 days (*n* = 166). Polysubstance use was defined as the use of 2 or more substances in the past 14 day

Substance use characteristics of the sample are depicted in Fig. 1. Alcohol was the most used substance with 73.5% reporting at least one drinking day in the past 14 days. Participants who used alcohol reported a mean of 5.01 drinking days (SD = 4.09) and 1.75 binge drinking days (SD = 3.07) over the past 14 days. Among participants who used alcohol, 32.5% reported drinking more drinks and 17.6% reported drinking more potent drinks since initiation of the stay-at-home order. Marijuana was used by 71.2% of the sample and tobacco was used by 41.6% of the sample in the past 14 days. Participants who smoked tobacco reported a mean of 11.56 smoking days (SD = 4.41) over the past 14 days and smoking a mean of 4.63 cigarettes per day (SD = 0.86) on smoking days. Illegal drug use, which does not include marijuana, was reported by 17.7% of the sample. Ecstasy/MDMA use was most common (11.1%), followed by powder cocaine (7.5%) and methamphetamine (2.2%). Use of crack-cocaine, GHB/GBL, ketamine, and heroin was uncommon (< 1% of the sample). In terms of polysubstance use, 11.9% reported no substance use, 17.3% used one substance, 63.7% used two or more substances in the past 14 days.

Complete bivariate analyses between sociodemographic characteristics and substance use are provided as a supplementary table (Table S1). Participants who were in a relationship were more likely to drink alcohol compared to participants who were single (Χ^2^ = 7.12, *p* = .008). Among those that drank alcohol, participants who reported an increased drinking frequency since initiation of the stay-at-home order were more likely to identify as male (Χ^2^ = 12.36, *p* = .002) and to not have a place to stay compared to those who reported a decreased drinking frequency (Χ^2^ = 0.93, *p* = .010). We did not observe differences in binge drinking or changes in the strength of drinks consumed. Participants who had a place to stay during the COVID-19 pandemic were more likely to use marijuana compared to those who did not have a place to stay (Χ^2^ = 8.93, *p* = .003). Participants who identified as gay were less likely to use illicit drugs compared to participants who identified as bisexual or something else (Χ^2^ = 8.35, *p* = .004), and participants who did not have an income during the COVID-19 pandemic were more likely to use illegal drugs compared to those who had an income (Χ^2^ = 7.48, *p* = .006). No differences were observed between participants who engaged in polysubstance use compared to participants who used less than two substances. Lockdown phase was not associated with substance use in bivariate analyses (*p* > 0.05).

Multivariable analyses are presented in Table 2. Participants who used marijuana had a prevalence of alcohol use 0.65 times greater than participants who did not use marijuana (APR = 1.65, 95% CI = 1.29, 2.11; *p* < .001). Among participants who used alcohol, participants who engaged in binge drinking had a prevalence of tobacco use 0.64 times greater than participants who did not engage in binge drinking (APR = 1.65; 95% CI = 1.13, 2.39, *p* = .010). Housing status was associated with changes in drinking frequency where participants who did not have housing during the COVID-19 pandemic were more likely to report an increase in their drinking frequency compared to those who had housing (APR = 1.73; 95% CI = 1.18, 2.54, *p* = .005). Marijuana use was more prevalent among participants whose education level was equal to or greater than a high school diploma compared to those with less than a high school diploma (APR = 1.69; 95% CI = 1.18, 2.42, *p* = .004) and among participants who did not have housing during the COVID-19 pandemic compared to those who had housing (APR = 1.22; 95% CI = 1.06, 1.40, *p* = .005). Participants who used alcohol had a prevalence of marijuana use 0.67 times greater than participants who did not use alcohol (APR = 1.67; 95% CI = 1.28, 2.18, *p* < .001) and participants who used tobacco had a prevalence of marijuana use 0.27 times greater than those who did not use tobacco (APR = 1.27; 95% CI = 1.08, 1.48, *p* = .003). Age was positively associated with illegal drug use (APR = 1.09; 95% CI = 1.03, 1.17, *p* = .006) and illegal drug use was 1.60 times prevalent among participants who used tobacco compared to participants who did not use tobacco (APR = 2.60; 95% CI = 1.34, 5.05, *p* = .005).

**Table 2:** Results of multivariable regression analyses examining the association between lockdown phase, sociodemographic characteristics, and substance use during the COVID-19 pandemic among participants in the N2 cohort, 2020

	Alcohol Use		Binge Drinking		Change in Drink Frequency				Change in Drink Strength			
					More (ref: same)		More (ref: fewer)		Stronger (ref: same)		Stronger (ref: weaker)	
	B	APR	B	APR	B	APR	B	APR	B	APR	B	APR
Reopening Phase (ref: restrictive phase)	− 0.14	0.87	0.37	1.45	0.03	1.03	−0.05	0.95	0.24	1.26	0.23	1.26
Age	−0.01	0.99	0.02	1.02	0.03	1.03	0.02	1.02	0.02	1.02	0.01	1.01
Male (ref: Transgender/Other)	0.18	1.19	−0.03	0.97	0.47	1.60	1.26	3.53*	0.44	1.56	0.09	1.09
Gay (ref: Bisexual/Other)	0.17	1.19*	0.17	1.18	0.16	1.17	−0.01	0.99	−0.06	0.94	0.28	1.32
Single (ref: In Relationship)	0.22	1.24*	−0.25	0.78	−0.25	0.78	0.08	1.09	−0.30	0.74	−0.35	0.71
HS Diploma (ref: less than HS diploma)	−0.27	0.76*	0.04	1.04	−0.11	0.90	−0.10	0.90	−0.18	0.83	0.26	1.29
Unemployment (ref: employed)	0.01	1.01	−0.24	0.79	0.33	1.39	−0.06	0.94	−0.43	0.65	−0.46	0.63
Income (ref: less than $20 K)	0.02	1.02	−0.12	0.88	0.11	1.11	0.04	1.04	−0.57	0.56	−0.08	0.93
Homelessness (ref: no)	−0.13	0.88	−0.18	0.84	−0.26	0.77	−0.10	0.91	−0.07	0.94	0.01	1.01
COVID-19 Income (ref: had income)	−0.12	0.89	−0.23	0.80	0.15	1.16	0.07	1.08	0.40	1.50	0.48	1.62
COVID-19 Insurance (ref: had insurance)	0.00	1.00	−0.25	0.78	−0.20	0.82	−0.08	0.92	−0.08	0.92	−0.18	0.83
COVID-19 Housing (ref: had housing)	−0.05	0.96	0.36	1.43	0.55	1.73*	0.16	1.18	0.29	1.34	0.34	1.40
Tobacco Use (ref: no use)	0.10	1.11	0.50	1.64**	0.16	1.17	−0.15	0.86	0.17	1.19	0.40	1.49
Marijuana Use (ref: no use)	0.50	1.65***	−0.10	0.91	0.54	1.72	0.52	1.68	1.05	2.87	0.01	1.01
Illegal Drug Use (ref: no use)	0.11	1.12	0.38	1.47*	0.11	1.12	0.14	1.15	0.28	1.32	0.21	1.23
	**Tobacco Use**		**Marijuana Use**				**Illegal Drug Use**			**Polysubstance Use**		
	B	APR	B		APR		B	APR		B	APR	
Reopening Phase (ref: restrictive phase)	0.06	1.06	0.08		1.08		−0.10	0.91		−0.07	0.94	
Age	0.04	1.04*	−0.02		0.98		0.09	1.09**		0.00	1.00	
Male (ref: Transgender/Other)	0.30	1.35	0.15		1.16		0.86	2.37		0.37	1.45	
Gay (ref: Bisexual/Other)	−0.21	0.81	0.03		1.03		−0.73	0.48*		−0.01	1.00	
Single (ref: In Relationship)	−0.12	0.89	0.06		1.06		0.20	1.22		0.20	1.22*	
HS Diploma (ref: less than HS diploma)	−0.06	0.94	0.53		1.69**		−0.94	0.39*		0.13	1.14	
Unemployment (ref: employed)	0.34	1.40	−0.07		0.94		0.20	1.22		0.17	1.18	
Income (ref: less than $20 K)	0.14	1.15	0.04		1.04		0.04	1.04		0.08	1.08	
Homelessness (ref: no)	0.20	1.23	0.19		1.21*		−0.49	0.62		0.25	1.28	
COVID-19 Income (ref: had income)	0.10	1.11	0.10		1.10		1.10	2.99		0.90	2.46	
COVID-19 Insurance (ref: had insurance)	−0.28	0.76	0.19		1.20*		0.26	1.30		0.12	1.13	
COVID-19 Housing (ref: had housing)	0.12	1.13	0.20		1.22**		−0.11	0.90		0.16	1.18	
Alcohol Use (ref: no use)	0.25	1.28	0.52		1.67***		0.52	1.68		–	–	
Tobacco Use (ref: no use)	–	–	0.24		1.27**		0.96	2.60***		–	–	
Marijuana Use (ref: no use)	0.70	2.02*	–		–		0.78	2.17		–	–	
Illicit Drug Use (ref: no use)	0.41	1.50*	0.15		1.16		–	–				

Note: * *p* ≤ .05; ** *p* ≤ .01; *** *p* ≤ .001. Abbreviations: *ref.* reference group, *HS* high school, *APR* adjusted prevalence ratio. Polysubstance use was defined as use of two or more substances in past 14 days

## Discussion

Substance use was common in our Chicago-based sample of Black cisgender SMM and transgender women during the initial peak of the COVID-19 pandemic. Approximately three of every four participants surveyed reported at least one drinking day and three of every four participants reported using marijuana at least once in the past 14 days. Tobacco use was less common, with two of every five participants reporting recent use, and approximately one in 10 participants reporting illegal drug use. Compared to previous research, alcohol and marijuana use rates appear elevated, whereas tobacco and illegal drug use are comparable. For example, in a pre-COVID-19 study of young Black sexual and gender minorities in Chicago, the prevalence of past three months use was 54.5% for alcohol, 46.1% for marijuana, and 19.5% for illegal drug use [26, 27]. Data from a pre-COVID-19 national probability sample of Black cisgender SMM indicated that 64.9% of these men used marijuana in their lifetime and 33.8% were current smokers [28]. Notably, recreational marijuana use was legalized in Chicago just months before the COVID-19 pandemic began so additional research is warranted to disaggregate the impact of COVID-19 on marijuana use from the impact of legalization.

Lockdown phase was not associated with substance use, despite concerns from previous research [4, 7]. Although lockdown phase was not associated with changes in drink quantity, 30.2% of participants who used alcohol reported drinking more since initiation of the stay-at-home order. For comparison, 26.0% of participants in a study of SMM in the US reported drinking more since the beginning of the COVID-19 pandemic [9]. This suggests that for a subset of the population, substance use increased following the stay-at-home orders and may have not decreased when these orders began to be lifted. We may have been underpowered to detect differences in this sample and future research with a larger sample would permit meaningful subgroup analyses (e.g., analyses specific to transgender women).

Most research indicates alcohol and marijuana use increased in the US population during the early months of the COVID-19 pandemic [29]. This is evident by the increase in sales of alcohol and marijuana compared to similar time frames in years prior to the COVID-19 pandemic [6, 30] and retrospective self-reports [31]. Factors associated with stay-at-home orders (e.g., social isolation, loneliness) may have contributed more to substance use than the issued orders [4]. However, most research examining other factors has produced mixed results [8, 29]. COVID-19 related stressors likely impacted substance use in this population by converging with existing social inequalities affecting Black communities [32]. Future research is needed that examines the impact of these converging factors on substance use [29]. Regardless of the cause, substance use may have been utilized to dampen emotional distress associated with the COVID-19 pandemic [3]. As the COVID-19 pandemic continues, increased screening for substance use disorders may be necessary for vulnerable communities.

Substance use was consistently associated with the use of other substances and, as a result, the rate of polysubstance use was high. Although previous research among cisgender SMM [33] has demonstrated significant positive associations between alcohol, tobacco, marijuana, and illegal drug use, less is known about these associations among Black SMM and transgender women. Consistent with this research, positive associations were observed between alcohol, tobacco, and marijuana use, and between tobacco and illegal drug use. Polysubstance use has been identified as a significant risk factor for HIV transmission among SMM and transgender women in the context of other HIV syndemic factors (e.g., depression, partner violence [19, 21, 34];). The high rate of polysubstance use in this sample is particularly concerning given the disproportionate impact the HIV epidemic has had on Black SMM and transgender women. Similar factors influencing COVID-19 outcomes in Black communities may also be impacting polysubstance use behavior [35]. As such, continued assessment of COVID-19 and polysubstance use is needed to understand predictors and patterns of polysubstance use, use severity, and prevent associated consequences.

The results from this study should be considered in the context of some limitations. First, substance use data were collected as part of a cross-sectional supplemental survey during the early months of the COVID-19 pandemic. As such, we were unable to compare rates of substance use over time in our sample and have relied on previously published studies for comparisons. Further, we assessed use at the initial peak of the COVID-19 pandemic and do not know how lasting the COVID-19 pandemic effects will be or if new variants will require reinstating restrictive mitigation strategies that prolong the effects of COVID-19 on substance use. Large-scale events, such as the COVID-19 pandemic, may increase rates of high-risk behaviors, including substance use [36]. However, research examining substance use during the early months of the COVID-19 pandemic has been largely inconsistent [8]. Longitudinal research is necessary to understand the true impact of COVID-19 on substance use [31]. Second, our measures of substance use provided little information on use frequency and use severity for marijuana and illegal drugs limiting the types of analyses we were able to perform with these data. We have plans to continue to monitor substance use in our sample and address these limitations as the city of Chicago continues to navigate COVID-19 related restrictions and we plan to publish these data in the future. Third, to reduce the likelihood of Type 1 error in our analyses, we utilized a more conservative significance threshold in hypothesis testing, and we may have overlooked smaller effects. A larger sample would have increased our power for detecting smaller effects and running subgroup analyses. Fourth, the sampling design limits the generalizability of our results. There has been significant variation in the response to COVID-19 across the US [3] and our results may not generalize to Black SMM and transgender women living in other locales, including other large urban cities.

## Conclusions

Rates of substance use were high among Black cisgender SMM and transgender women in Chicago during the initial peak of the COVID-19 pandemic. Additional research is needed to identify the impact of the COVID-19 pandemic on substance use severity in this population. Further, continued monitoring of substance use behaviors, including polysubstance use, and screening for substance use disorders is needed as the COVID-19 pandemic continues given the various negative health consequences associated with substance use.

## Supplementary Information

**Additional file 1: Table S1.** cont. Rates of substance use by lockdown phase and across sociodemographic characteristics during the Covid-19 pandemic among participants in the N2 cohort, 2020.

## Data Availability

The datasets used and/or analysed during the current study are available from the corresponding author on reasonable request.

## Abbreviations

SMM: Sexual minority men
APR: Adjusted prevalence ratio
