# Supplementary material for: Polysubstance use in a community sample of Black cisgender sexual minority men and transgender women in Chicago during initial COVID-19 pandemic peak
Source: Subst Abuse Treat Prev Policy. 2022 Jan 28;17:4. doi: 10.1186/s13011-022-00433-x (PMC8796750; doi:10.1186/s13011-022-00433-x)
Supplement: Supplementary file 1 — Additional file 1: Table S1. cont. Rates of substance use by lockdown phase and across sociodemographic characteristics during the Covid-19 pandemic among participants in the N2 cohort, 2020. [file 13011_2022_433_MOESM1_ESM.docx]

| Table S1  Rates of substance use by lockdown phase and across sociodemographic characteristics during the Covid-19 pandemic among participants in the N2 cohort, 2020. | | | | | | | | | | | | |
| --- | --- | --- | --- | --- | --- | --- | --- | --- | --- | --- | --- | --- |
|  | Total | | Alcohol Use | | | Binge Drinking | | | Tobacco Use | | |  |
|  |  |  | Yes (n = 166) | | | Yes (n = 75) | | | Yes (n = 94) | | |  |
|  | n | % | n | % | *χ²* | n | % | *χ²* | n | % | *χ²* |  |
| Lockdown phase |  |  |  |  |  |  |  |  |  |  |  |  |
| Restrictive Phase | 77 | 34.1 | 61 | 36.7 | 1.99 | 24 | 32.0 | 1.53 | 30 | 31.9 | 0.33 |  |
| Reopening Phase | 149 | 65.9 | 105 | 63.3 |  | 51 | 68.0 |  | 64 | 68.1 |  |  |
| Age |  |  |  |  |  |  |  |  |  |  |  |  |
| 16-22 | 51 | 22.7 | 39 | 23.6 | 0.81 | 18 | 24.0 | 1.01 | 17 | 18.1 | 2.66 |  |
| 23-24 | 49 | 21.8 | 36 | 21.8 |  | 14 | 18.7 |  | 20 | 21.3 |  |  |
| 25-28 | 63 | 28.0 | 47 | 28.5 |  | 21 | 28.0 |  | 27 | 28.7 |  |  |
| 29-36 | 62 | 27.6 | 43 | 26.1 |  | 22 | 29.3 |  | 30 | 31.9 |  |  |
| Gender |  |  |  |  |  |  |  |  |  |  |  |  |
| Male | 196 | 86.7 | 148 | 89.2 | 3.21 | 8 | 10.7 | 0.00 | 82 | 87.2 | 0.04 |  |
| Trans Feminine/Other | 30 | 13.3 | 18 | 10.8 |  | 67 | 89.3 |  | 12 | 12.8 |  |  |
| Sexual Orientation |  |  |  |  |  |  |  |  |  |  |  |  |
| Gay | 131 | 58.0 | 103 | 62.0 | 4.28* | 46 | 61.3 | 0.01 | 46 | 48.9 | 5.38* |  |
| Bisexual/Straight/Something Else | 95 | 42.0 | 63 | 38.0 |  | 29 | 38.7 |  | 48 | 51.1 |  |  |
| Relationship Status |  |  |  |  |  |  |  |  |  |  |  |  |
| In Relationship | 138 | 61.1 | 110 | 66.3 | 7.12** | 47 | 62.7 | 0.88 | 59 | 62.8 | 0.20 |  |
| Single | 88 | 38.9 | 56 | 33.7 |  | 28 | 37.3 |  | 35 | 37.2 |  |  |
| Education |  |  |  |  |  |  |  |  |  |  |  |  |
| Less than HS Diploma | 23 | 10.2 | 17 | 10.2 | 0.00 | 9 | 12.0 | 0.49 | 11 | 11.7 | 0.41 |  |
| HS Diploma | 203 | 89.8 | 149 | 89.8 |  | 66 | 88.0 |  | 83 | 88.3 |  |  |
| Employment |  |  |  |  |  |  |  |  |  |  |  |  |
| Employed | 130 | 57.5 | 98 | 59.0 | 0.59 | 46 | 61.3 | 0.24 | 46 | 48.9 | 4.86* |  |
| Unemployed | 96 | 42.5 | 68 | 41.0 |  | 29 | 38.7 |  | 48 | 51.1 |  |  |
| Income |  |  |  |  |  |  |  |  |  |  |  |  |
| Less than 20K | 140 | 62.5 | 99 | 60.0 | 1.67 | 43 | 57.3 | 1.67 | 57 | 60.6 | 0.24 |  |
| 20K plus | 84 | 37.5 | 66 | 40.0 |  | 32 | 42.7 |  | 37 | 39.4 |  |  |
| Homelessness (past 12 months) |  |  |  |  |  |  |  |  |  |  |  |  |
| No | 169 | 74.8 | 128 | 77.1 | 1.80 | 59 | 78.7 | 0.16 | 65 | 69.1 | 2.71 |  |
| Yes | 57 | 25.2 | 38 | 22.9 |  | 16 | 21.3 |  | 29 | 30.9 |  |  |
| Income |  |  |  |  |  |  |  |  |  |  |  |  |
| No Income | 171 | 75.7 | 122 | 73.5 | 1.60 | 21 | 28.0 | 0.19 | 77 | 81.9 | 0.08 |  |
| Had Income | 55 | 24.3 | 44 | 26.5 |  | 54 | 72.0 |  | 17 | 18.1 |  |  |
| Health Insurance |  |  |  |  |  |  |  |  |  |  |  |  |
| No Insurance | 48 | 21.6 | 37 | 22.7 | 0.42 | 14 | 19.2 | 0.86 | 18 | 19.6 | 0.39 |  |
| Had Insurance | 174 | 78.4 | 126 | 77.3 |  | 59 | 80.8 |  | 74 | 80.4 |  |  |
| Place to Stay |  |  |  |  |  |  |  |  |  |  |  |  |
| No place to stay | 55 | 24.3 | 30 | 18.1 | 0.01 | 16 | 21.3 | 1.05 | 21 | 22.6 | 2.02 |  |
| Had place to stay | 171 | 75.7 | 136 | 81.9 |  | 59 | 78.7 |  | 72 | 77.4 |  |  |
| Note: * p ≤ .05; ** p ≤ .01; *** p ≤ .001. Binge drinking analyses only included participants who drank at least one day in past 14 days. | | | | | | | | | | | |  |

| Table S1 cont.  Rates of substance use by lockdown phase and across sociodemographic characteristics during the Covid-19 pandemic among participants in the N2 cohort, 2020. | | | | | | | | | | | |
| --- | --- | --- | --- | --- | --- | --- | --- | --- | --- | --- | --- |
|  | Total | | Marijuana Use | | | Illegal Drug Use | | | Polysubstance Use | | |
|  |  |  | Yes (n =161) | | | Yes (n = 40) | | | Yes (n =160) | | |
|  | n | % | n | % | *χ²* | n | % | *χ²* | n | % | *χ²* |
| Lockdown phase |  |  |  |  |  |  |  |  |  |  |  |
| Restrictive Phase | 77 | 34.1 | 53 | 32.9 | 0.33 | 13 | 32.5 | 0.05 | 56 | 35.0 | 0.21 |
| Reopening Phase | 149 | 65.9 | 108 | 67.1 |  | 27 | 67.5 |  | 104 | 65.0 |  |
| Age |  |  |  |  |  |  |  |  |  |  |  |
| 16-22 | 51 | 22.7 | 38 | 23.6 | 3.16 | 6 | 15.0 | 3.08 | 35 | 20.6 | 3.06 |
| 23-24 | 49 | 21.8 | 37 | 23.0 |  | 8 | 20.0 |  | 34 | 20.0 |  |
| 25-28 | 63 | 28.0 | 47 | 29.2 |  | 11 | 27.5 |  | 50 | 29.4 |  |
| 29-36 | 62 | 27.6 | 39 | 24.2 |  | 15 | 37.5 |  | 51 | 30.0 |  |
| Gender |  |  |  |  |  |  |  |  |  |  |  |
| Male | 196 | 86.7 | 143 | 88.8 | 2.13 | 36 | 90.0 | 0.45 | 143 | 89.4 | 3.34 |
| Trans Feminine/Other | 30 | 13.3 | 18 | 11.2 |  | 4 | 10.0 |  | 17 | 10.6 |  |
| Sexual Orientation |  |  |  |  |  |  |  |  |  |  |  |
| Gay | 131 | 58.0 | 93 | 57.8 | 0.01 | 15 | 37.5 | 8.35** | 92 | 57.5 | 0.05 |
| Bisexual/Straight/Something Else | 95 | 42.0 | 68 | 42.2 |  | 25 | 62.5 |  | 68 | 42.5 |  |
| Relationship Status |  |  |  |  |  |  |  |  |  |  |  |
| In Relationship | 138 | 61.1 | 103 | 64.0 | 0.20 | 27 | 67.5 | 0.85 | 56 | 35.0 | 3.57 |
| Single | 88 | 38.9 | 58 | 36.0 |  | 13 | 32.5 |  | 104 | 65.0 |  |
| Education |  |  |  |  |  |  |  |  |  |  |  |
| Less than HS Diploma | 23 | 10.2 | 13 | 8.1 | 2.71 | 7 | 17.5 | 2.85 | 15 | 9.4 | 0.39 |
| HS Diploma | 203 | 89.8 | 148 | 91.9 |  | 33 | 82.5 |  | 145 | 90.6 |  |
| Employment |  |  |  |  |  |  |  |  |  |  |  |
| Employed | 130 | 57.5 | 92 | 57.1 | 0.03 | 20 | 50.0 | 1.13 | 87 | 54.4 | 2.22 |
| Unemployed | 96 | 42.5 | 69 | 42.9 |  | 20 | 50.0 |  | 73 | 45.6 |  |
| Income |  |  |  |  |  |  |  |  |  |  |  |
| Less than 20K | 140 | 62.5 | 99 | 61.9 | 0.09 | 23 | 57.5 | 0.52 | 98 | 61.6 | 0.18 |
| 20K plus | 84 | 37.5 | 61 | 38.1 |  | 17 | 42.5 |  | 61 | 38.4 |  |
| Homelessness (past 12 months) |  |  |  |  |  |  |  |  |  |  |  |
| No | 169 | 74.8 | 115 | 71.4 | 3.33 | 31 | 77.5 | 0.19 | 119 | 74.4 | 0.05 |
| Yes | 57 | 25.2 | 46 | 28.6 |  | 9 | 22.5 |  | 41 | 25.6 |  |
| Income |  |  |  |  |  |  |  |  |  |  |  |
| No Income | 171 | 75.7 | 127 | 78.9 | 3.15 | 37 | 92.5 | 7.48** | 125 | 78.1 | 1.80 |
| Had Income | 55 | 24.3 | 34 | 21.1 |  | 3 | 7.5 |  | 35 | 21.9 |  |
| Health Insurance |  |  |  |  |  |  |  |  |  |  |  |
| No Insurance | 48 | 21.6 | 40 | 25.2 | 4.13* | 10 | 26.3 | 0.60 | 17 | 12.4 | 1.20 |
| Had Insurance | 174 | 78.4 | 119 | 74.8 |  | 28 | 73.7 |  | 120 | 87.6 |  |
| Place to Stay |  |  |  |  |  |  |  |  |  |  |  |
| No place to stay | 55 | 24.3 | 37 | 23.1 | 8.93** | 8 | 20.5 | 0.17 | 33 | 20.8 | 2.33 |
| Had place to stay | 171 | 75.7 | 123 | 76.9 |  | 31 | 79.5 |  | 126 | 79.2 |  |
| Note: * p ≤ .05; ** p ≤ .01; *** p ≤ .001. Polysubstance use defined as use of two or more substances in past 14 days. | | | | | | | | | | | |
